# The Effect of a Neuronal Nitric Oxide Synthase Inhibitor on Neurovascular Regulation in Humans

**DOI:** 10.1161/CIRCRESAHA.122.321631

**Published:** 2022-11-09

**Authors:** Kevin O’Gallagher, Ryan E. Rosentreter, Jan Elaine Soriano, Ali Roomi, Saqib Saleem, Tyler Lam, Roman Roy, Grant R. Gordon, Satish R. Raj, Philip J. Chowienczyk, Ajay M. Shah, Aaron A. Phillips

**Affiliations:** 1 School of Cardiovascular and Metabolic Medicine & Sciences, King’s College London British Heart Foundation Centre of Research Excellence, London, UK (K.O., A.R., R.R., P.J.C., A.M.S.).; 2 NIHR Biomedical Research Centre, Clinical Research Facility, Guy’s and St Thomas NHS Foundation Trust, London, UK (K.O., A.R., P.J.C., A.M.S.).; 3 Departments of Physiology and Pharmacology, Clinical Neurosciences, Cardiac Sciences, Hotchkiss Brain Institute, Libin Cardiovascular Institute of Alberta, Cumming School of Medicine, University of Calgary, Alberta, Canada (R.E.R, J.E.S., T.L., G.R.G., S.R.R., A.A.P.).; 4 Department of Electrical and Computer Engineering, COMSATS University, Sahiwal, Pakistan (S.S.).

**Keywords:** neurovascular coupling, nitric oxide

## Abstract

Neurovascular coupling (NVC) is a key process in cerebral blood flow regulation. NVC ensures adequate brain perfusion to changes in local metabolic demands. Neuronal nitric oxide synthase (nNOS) is suspected to be involved in NVC; however, this has not been tested in humans. Our objective was to investigate the effects of nNOS inhibition on NVC in humans.

**Methods:**

We performed a 3-visit partially randomized, double-blinded, placebo-controlled, crossover study in 12 healthy subjects. On each visit, subjects received an intravenous infusion of either S-methyl-L-thiocitrulline (a selective nNOS-inhibitor), 0.9% saline (placebo control), or phenylephrine (pressor control). The NVC assessment involved eliciting posterior circulation hyperemia through visual stimulation while measuring posterior and middle cerebral arteries blood velocity.

**Results:**

nNOS inhibition blunted the rapidity of the NVC response versus pressor control, evidenced by a reduced initial rise in mean posterior cerebral artery velocity (−3.3% [−6.5, −0.01], *P*=0.049), and a reduced rate of increase (ie, acceleration) in posterior cerebral artery velocity (slope reduced −4.3% [−8.5, −0.1], *P*=0.045). The overall magnitude of posterior cerebral artery response relative to placebo control or pressor control was not affected. Changes in BP parameters were well-matched between the S-methyl-L-thiocitrulline and pressor control arms.

**Conclusions:**

Neuronal NOS plays a role in dynamic cerebral blood flow control in healthy adults, particularly the rapidity of the NVC response to visual stimulation. This work opens the way to further investigation of the role of nNOS in conditions of impaired NVC, potentially revealing a therapeutic target.

**Novelty and Significance**

**What Is Known?**

- Neurovascular coupling (NVC) is an important mechanism in neurovascular regulation
- Nitric oxide (NO) is an important mediator of NVC responses
- nNOS (neuronal nitric oxide synthase; as a source of NO) is known to play a role in cerebrovascular regulation, but its precise role in NVC is unclear

**What New Information Does this Article Contribute?**

- nNOS (as a source of NO) has a role in the regulation of the rapidity of NVC responses
- nNOS does not affect the magnitude of NVC responses

Neurovascular coupling (NVC) is a key process in cerebral blood flow regulation, ensuring adequate brain perfusion to changes in local metabolic demands. NO is known to be involved in NVC responses, but the contribution of specific nitric oxide synthase (nNOS) isoforms was previously unclear. This is the first human study to demonstrate the role for nNOS in the regulation of NVC responses, particularly the rapidity of the NVC response to visual stimulation.

**In This Issue, see p 949**

**Meet the First Author, see p 950**

The central nervous system’s voracious energy consumption and lack of substrate storage capacity necessitates sophisticated cerebral blood flow regulation to ensure appropriate perfusion. One of the primary regulatory pathways involved in neurovascular control is neurovascular coupling (NVC).^1^

NVC represents the relationship between neuronal activity and local central nervous system blood flow, allowing the brain to match regional perfusion levels to the metabolic demand. Dysfunctional NVC is associated with early vascular cognitive impairment,^2^ and has been identified in neurological diseases such as Alzheimer’s disease and spinal cord injury^3,4^ as well as cardiovascular pathologies such as hypertension and atrial fibrillation.^5–7^ Briefly, modulated neuronal activity, and the ensuing changes in glutamate levels, cause changes in local blood flow by adjusting vascular tone via pial arteries, penetrating arterioles, and pericytes enveloped around capillaries.^8,9^ These adjustments in vascular tone are thought to be elicited through rapid and transient direct neuronal-to-vascular cascades, as well as indirect slower and sustained astrocyte-mediated pathways. Although NVC is a well-established physiological response, with altered blood pressure playing a key role, a comprehensive mechanistic understanding is lacking. Studies in anesthetized animals indicate that more than half of the neurovascular cascade is driven by nitric oxide (NO), a powerful vasodilator that activates soluble guanylate cyclase in vascular smooth muscle.^10^ Emerging preclinical work indicates that the early phase direct neuronal-to-vascular cascade is modulated by neuronal production of NO through neuronal nitric oxide synthase (nNOS).^10^ It is not currently clear if this effect is translatable to humans. Although it is well-established that NO is involved in the regulation of basal central nervous system blood flow in humans,^11,12^ studies have not to date identified the individual NOS isoform that is responsible, nor its effect on NVC.

The role of nNOS in NVC has not previously been studied in humans, but it is important to define since many cerebrovascular disorders are associated with abnormalities in this fundamental regulatory mechanism. Therefore, the mechanisms underpinning NVC responses could represent novel therapeutic targets for these conditions. Accordingly, in addition to furthering understanding of patho-mechanisms and the interplay of cardiovascular and neurovascular disease, there is clear translational potential in identifying the mechanisms of dysfunctional NVC responses.

In this study, we have undertaken the first direct investigation of the role of nNOS in NVC in healthy humans, using a 3-visit double-blinded, placebo-controlled, crossover study design. We tested NVC during intravenous administration of a well-characterized selective nNOS inhibitor, S-methyl-L-thiocitrulline (SMTC). The responses during SMTC infusion were compared with those during a control condition where blood pressure was matched using titrated phenylephrine or to saline placebo.

## Methods

### Data Availability

The data that support the findings of this study are available from the corresponding author upon reasonable request.

### Ethics Approval

This study adhered to the standards outlined in the Declaration of Helsinki. Ethical approval for this study was obtained from the Conjoint Health Research Ethics Board (REB19-1613) at the University of Calgary and the London-Dulwich Research Ethics Committee (18/LO/2064). Written informed consent was obtained from all volunteers prior to commencing any protocol-related procedures. The study was performed at a single site; the NIHR Biomedical Research Centre, St Thomas’ Hospital, Guy’s and St Thomas’ NHS Foundation Trust, London, UK.

### Participants

A total of 12 participants completed all 3 visits (5 men, 7 women, mean age 27±7 years). One participant had a vasovagal response during experimental set-up and therefore is excluded from the final analysis (see Figure S1 for recruitment flow chart and Table S1 for participant characteristics). Participants were healthy adult volunteers with normal blood pressure (systolic blood pressure <140mmg and diastolic blood pressure <90 mm Hg) without any recent illness, or regular systemic medication (other than the oral contraceptive pill). Exclusion criteria included altered circadian rhythms (eg, shift workers); active menstruation, pregnancy, or breastfeeding; current/past neurological or psychiatric diagnosis; use of recreational drugs within the last 12 months; current or regular opioid medications. Participants were required to abstain from a number of agents prior to each testing visit: alcohol (24 hours), caffeine (12 hours), NSAIDs/paracetamol (24 hours), tobacco/nicotine (4 hours).

### Protocol

We performed a 3-visit randomized, double-blinded, placebo- and pressor-controlled, crossover study to assess the effect of intravenous administration of S-methyl-L-thiocitrulline (SMTC, Merck Millipore, USA), an nNOS inhibitor, on measures of NVC. Intravenous infusion of SMTC is associated with a rise in mean arterial pressure (MAP) due to systemic nNOS inhibition.^13,14^ Therefore, given that blood pressure plays a strong role in NVC,^15–17^ in addition to a placebo control (0.9% saline), we also used a pressor control (phenylephrine, Amdipharm UK Ltd, an ɑ_1_ adrenoceptor agonist). SMTC is a synthetic L-arginine analogue strongly selective for nNOS versus endothelial nitric oxide synthase (eNOS).^18,19^ Rodent studies suggest that SMTC is 17-times more specific for nNOS in brain tissue than endothelial nitric oxide synthase in vascular endothelium.^20^ SMTC also crosses the blood brain barrier, as demonstrated through use of ^11^C-labeled SMTC in rat and primate models.^21^ SMTC was prepared to Good Manufacturing Practices standard for human use by Guy’s and St Thomas’ NHS Foundation Trust aseptic pharmacy. SMTC was infused at a dose of 3.0 µmol/kg for 10 minutes as a bolus, followed by 0.05 µmol/kg/min maintenance dose until the protocol was completed.^13,14^ The preparation of the SMTC infusion was calculated based on weight to allow for a standard rate of infusion for all participants (ie, 2 ml/min bolus followed by 1 ml/min maintenance infusion). The placebo control was infused at a rate identical to the SMTC. The pressor control was infused at a dose of 25–100 µg/min at a rate titrated to achieve a rise in MAP corresponding to that seen with SMTC (~7 mm Hg from our previous work).^13,14^ During the pressor control condition, we aimed to match MAP to the SMTC condition; therefore randomization between these conditions was not possible. However, at all points, the participants and the members of the research team performing NVC data collection remained fully blinded to the contents of the infusion. There was a minimum of 48 hours washout between study visits.

### Physiological Measures

Brachial blood pressure was obtained by standard noninvasive oscillometric methods (Intellivue, Phillips, UK). Finger plethysmography (Finometer NOVA, Finapres Medical Systems, The Netherlands) was used to provide estimations of the following hemodynamic variables: blood pressure, systemic vascular resistance (SVR), and cardiac output (CO). Heart rate was measured by placement of 3 electrocardiogram electrodes in a 3-lead bipolar arrangement and collected at a sampling rate of 1000 Hz. Cerebral blood flow velocity was assessed using 2 MHz transcranial Doppler probes (DWL DopplerBox X, Compumedics, Singen, Germany) inserted into an adjustable headpiece and positioned bilaterally against the temporal bones to insonate the middle cerebral artery (MCA) on the 1 side and posterior cerebral artery on the other as previously described.^22^ Cerebral blood flow velocities were assessed and recorded using software (QL Monitoring Software, version 3.5.5). End-tidal carbon dioxide and oxygen levels were monitored via the RespirAct, (Thornhill Research, Toronto, Canada). The breathing circuit was connected to the participant via a soft plastic mask custom-fitted to each participant. The mask was sealed to the participants face using transparent dressing film (Tegaderm Film, 1626W, 3M Healthcare, St. Paul, MN).

On each visit, pre- and postdrug assessments of NVC were performed. The standardized NVC assessment involved using a visual task to activate the visual cortex, resulting in hyperemia in the posterior circulation. This involved 10 cycles of 30 seconds with eyes closed, followed by 30 seconds of eyes open where the participant tracked a pendulum moving laterally, back and forth across the screen (repeated x-plane pattern, 3.5cm radius solid white circle on black background) on a 24-inch computer monitor, with the participant seated in an upright posture approximately 0.75 m from the computer monitor. This visual stimulus has been shown to be the most selective for the posterior circulation in in-human NVC testing.^23^ The research team members performing data collection of NVC responses were blinded to the contents of the infusion (placebo, SMTC, or phenylephrine), as were the volunteers. A separate member of the research team was responsible for preparation and administration of the infusion. In our hands, the test-retest reliability of NVC assessments is 0.95 (R^2^ = 0.91, *P*=3.4×10^6^; See Figure S2).

### *Neurovascular* Coupling Analysis

NVC data were analyzed through iNVC software (Version 2.2; Innovate Calgary, Calgary, Alberta, Canada). This software automatically analyzes NVC data collected on a variety of data acquisition systems, calculating the classical metrics characterizing the hemodynamic response, as well as proprietary iNVC summary measures that capture the key features (rapidity [iRate1/2], amplitude [iAmplitude1/2], pulsatility [iPulsatility]) within the neurovascular coupling response, and are stable within and across individuals.^24^ All hemodynamic variables were sampled at 1000 Hz and extracted on a beat-by-beat basis. All NVC metrics were calculated as absolute or percent change relative to the 15−5 seconds prior to eyes open. Each participant’s NVC metrics were calculated as the average of all included trials from the 10 cycles of visual task for each condition. In addition to assessing PCA velocities and associated metrics across the entire 30 second period of the NVC response, to assess the effect of nNOS on rapidity of NVC responses, we also measured PCA velocity responses during the first 5 seconds, when the rate of change of PCA velocity is highest.

MAP was calculated from systolic blood pressure and diastolic blood pressure as ([⅓ systolic blood pressure] + [⅔ diastolic blood pressure]). Mean MCA velocity (MCA_mean_) was calculated in a similar fashion using MCA max velocity (MCA_max_) and MCA minimum velocity (MCA_min_) as ((⅓ MCA_max_) + (⅔ MCA_min_)). Mean PCA velocity was calculated in this exact fashion. Cerebrovascular conductance through the MCA (MCA_CVC_) was calculated as MCA_mean_/MAP, and cerebrovascular resistance through the MCA (MCA_CVR_) was taken as the inverse of this relationship. Cerebrovascular conductance and resistance values for PCA were calculated in this exact fashion.

Please see the Major Resources Table in the Supplemental Materials.

### Statistical Analysis

Statistical analysis was carried out in accordance with the American Heart Association Recommendations for Statistical Reporting in Cardiovascular Medicine.^25^ Normality was assessed by applying the Shapiro-Wilk test. Comparisons between all 3 conditions used a 1-way ANOVA, with a Tukey HSD post-hoc for parametric data and Dunn post-hoc for nonparametric data. No experiment-wide multiple test correction was applied. *P<*0.05 was considered statistically significant. Parametric data are reported as mean±SEM. Nonparametric data are reported as median [interquartile range]. Outlier data were retained unless felt to be a measurement error.

## Results

### Resting Steady-State Hemodynamics

There were no statistically significant differences in steady-state pre-intervention hemodynamic variables between study visits (see Table 1). MAP was elevated to a similar extent by SMTC or pressor control when compared with placebo control (Table 1). Heart rate was decreased with SMTC and pressor control compared with placebo control. Stroke volume (SV) decreased with SMTC, relative to placebo control, whereas SV was increased by pressor control. Cardiac output decreased with SMTC, which was different compared with both placebo control (−1.2 L/min [0.3, 2.1] (mean [95% CI]), *P*=0.0097 and pressor control (−1.4 L/min [−2.0, −0.8], *P*=3.2×10^−^^4^). In summary, with the exception of SV, cardiac output, and systemic vascular resistance, the hemodynamic effects of SMTC and pressor control were similar, compared with the placebo control condition.

**Table 1.**
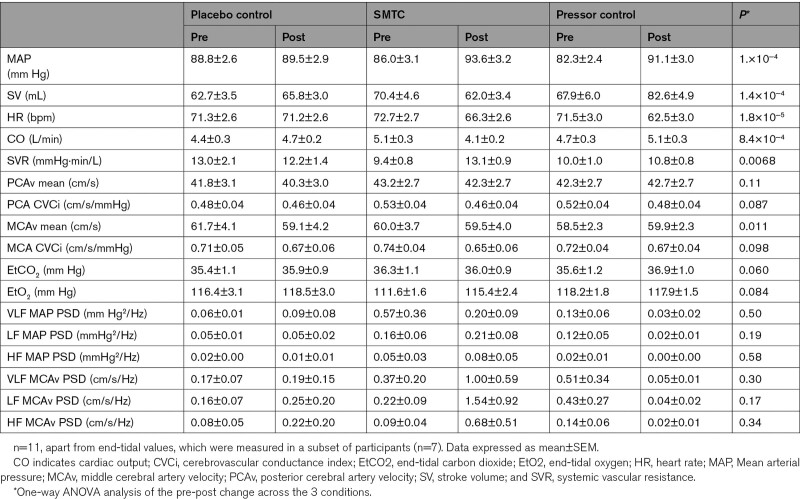
Steady-State Data

There were no statistically significant differences in baseline steady-state cerebrovascular hemodynamic variables between study visits (Table 1). There was no statistically significant difference between groups for mean blood velocity in the PCA or in cerebrovascular conductance for either PCA or MCA (Table 1). See Figure 2 for steady-state cerebrovascular hemodynamics. SMTC had no statistically significant effect on mean velocity in either the MCA or PCA, when compared with pressor control or placebo control, but did show a statistically significant increase in resistance in the MCA compared with placebo (Figure 2D, *P*=0.0085). However, the MCA conductance index with SMTC was comparable to that seen in the pressor control group (Table 1), suggesting that the increase in resistance with STMC represented an autoregulatory response.

**Figure 1.**
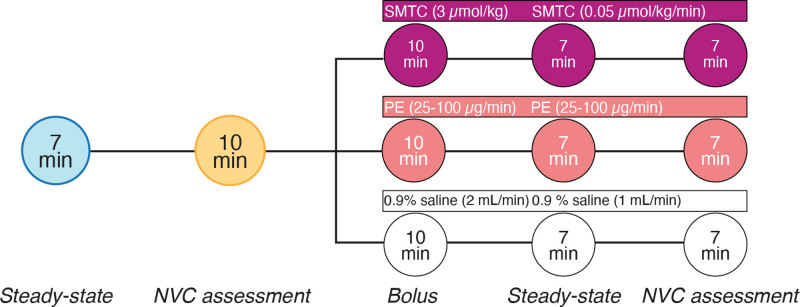
**Study protocol.** Each participant completed 3 separate visits a minimum of 48 hours apart. The protocol for each visit was randomized. On each test day, measurements of hemodynamics, end tidal oxygen and carbon dioxide, and cerebral blood flow velocities were obtained for a steady-state period and NVC assessment prior to drug or placebo administration. NVC indicates neurovascular coupling; PE, phenylephrine; and SMTC, S-methyl-L-thiocitrulline.

**Figure 2.**
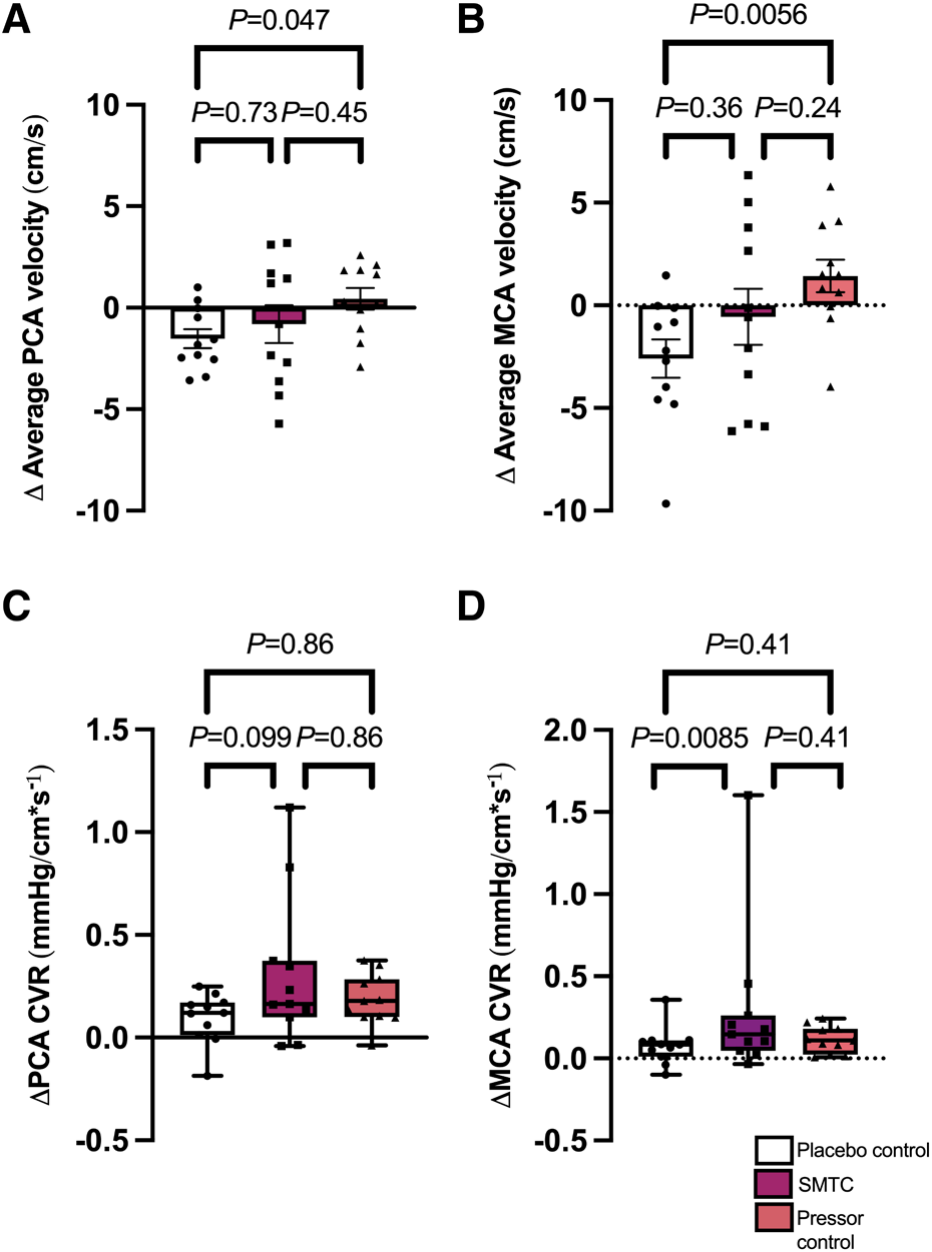
**Changes in steady-state cerebral hemodynamics between conditions.** Changes in cerebral hemodynamic measurements during maintenance infusion of SMTC, phenylephrine, or saline placebo. A, Average posterior cerebral artery (PCA) velocity. B, Average middle cerebral artery (MCA) velocity. C, PCA cerebrovascular resistance (CVR). D, MCA CVR. S-methyl-L-thiocitrulline (SMTC). Analyzed by 1-way ANOVA with Tukey post hoc (A, B) or Friedman test with Dunn multiple comparison test (C, D). Data presented as mean±SEM (A, B) or median (IQR) (C, D). n=11.

### Model of NVC

Across baseline conditions, mean PCA velocity increased from 38.6±1.3 to 45.6±1.5 cm/s during the eyes open period. Pre-intervention, there were no statistically significant differences in the PCA mean velocity response and peak velocity response to visual stimulation between conditions (*P*=0.62, *P*=0.82), nor the time to peak response (*P*=0.27). Additionally, metrics of rapidity (ie, slope in the initial 5 seconds and the maximum slope) did not show statistically significant differences across baseline conditions (*P=*0.81, *P=*0.65).

### Rapidity of the NVC Response Was Reduced With nNOS Inhibition

**Figure 3.**
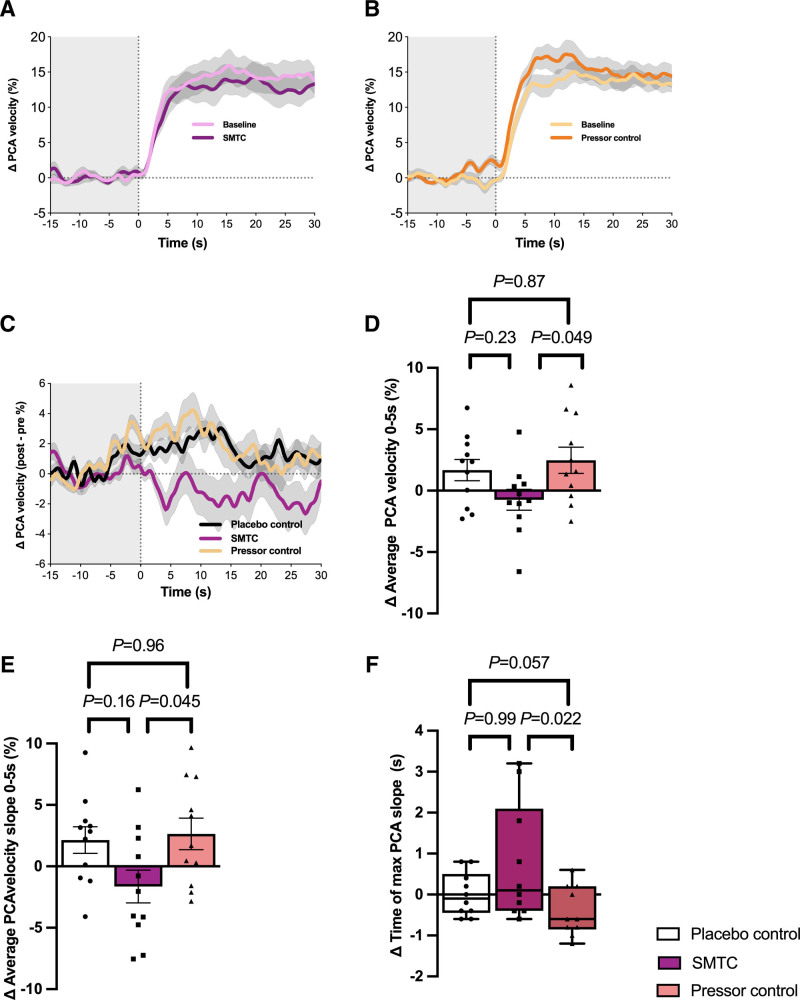
**Changes in PCA velocity during neurovascular coupling response.** A, B, Percentage change in PCA velocity in response to SMTC and pressor control respectively. C, Percentage change in PCA response (post-pre) for SMTC, placebo control and pressor control. D, Percentage change in average PCA velocity during first 5 seconds of NVC response. E, Percentage change in average PCA velocity slope during first 5 seconds of NVC response. F, Percentage change in time to maximal PCA velocity slope. In Figures D–F, white bars represent placebo control, purple bars represent SMTC, peach bars represent pressor control. Posterior cerebral artery (PCA), S-methyl-L-thiocitrulline (SMTC). Analyzed by 1-way ANOVA with Tukey post-hoc test (A–E) or Friedman test with Dunn’s multiple comparison test (F). Data presented as mean±SEM (D, E) or median (IQR) (F). Grey shadows in A–C represent SEM n=11 for A–E, n=10 for F (the data points for volunteer 2 have been removed from F analysis due to an extreme outlier for volunteer 2 placebo data point that was felt to be a measurement error).

Considering the entire 30 second period of the NVC response, there was no statistically significant overall difference in the change in PCA velocity from SMTC compared with either placebo control (*P*=0.46) or pressor control (*P*=0.32) (Figure 3C). There was, however, a statistically significant difference in PCA response during the first 5 seconds of the NVC cycle with the peak difference in ΔPCA velocity seen following SMTC infusion: −5.5% [−9.3, −1.7] (mean [95% CI]), (*P*=0.0013) versus pressor control and −3.7% [−7.5, −0.01], (*P*=0.026) versus placebo control, with both maximal changes seen at T=4 seconds (Figure 3C). Considering the first 5 seconds of the NVC cycles as a whole, both the mean PCA velocity (−3.3% [−6.5, −0.01], *P*=0.049, Figure 3D) and the rate of change of PCA velocity (−4.3%, [−8.5, −0.1], *P*=0.045, Figure 3E) were significantly decreased for SMTC versus pressor control. The time to maximal slope was significantly increased for SMTC versus pressor control (+0.1s [−0.4, 2.1] (median [IQR]) versus −0.6s [−0.9, 0.2], *P*=0.022, Figure 3F). Further analysis found that sex hemodynamic conditions had no statistically significant effect on the rapidity of NVC response (Figure S3). Trends in PCA velocity changes remained consistent when corrected for changes in cardiac output and systemic vascular resistance (Figure S4).

**Figure 4.**
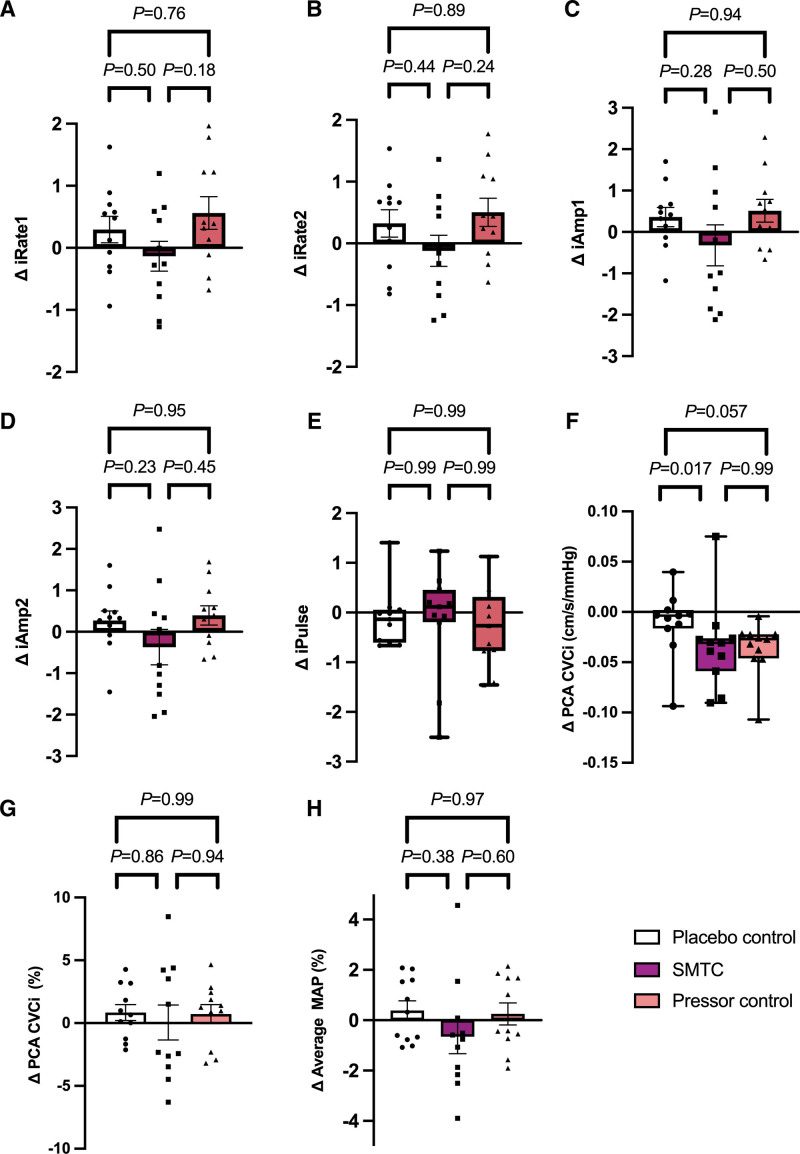
**Neurovascular coupling response.** Change in iNVC summary metrics of rapidity (A, B), amplitude (C, D) and pulsatility (E) of response between SMTC and control conditions. F, G, comparisons of absolute and percent PCA conductance between SMTC and control conditions. N, Changes in MAP during the NVC response between SMTC and control conditions. Posterior cerebral artery (PCA), S-methyl-L-thiocitrulline (SMTC), mean arterial pressure (MAP), cerebrovascular conductance index (CVCi). Comparisons between SMTC and control conditions were conducted using 1 way ANOVA with Tukey post hoc test (A–D, F-–H) or Friedman test with Dunn’s multiple comparison test (E). Data presented as mean±SEM (A–D, G, H) or median (IQR) (E and F). n=11.

There was no statistically significant difference in either the average or peak PCA mean velocity response with SMTC compared with pressor control (*P*=0.28, *P*=0.28 respectively). Neither was there a statistically significant difference between SMTC and pressor control in terms of iAmplitude1 and iAmplitude2 (*P*=0.41, *P*=0.40). See Figure 4 for NVC analysis between SMTC and pressor control. In summary, the rate of increase in the initial NVC response following eyes-open was reduced during SMTC when compared with pressor control; however, the amplitudinal response was not affected by SMTC.

## Discussion

Our goal was to interrogate the role of nNOS in NVC in healthy humans. To rigorously test this, we used a pre-post interventional design, where we matched the pressor responses to systemic nNOS inhibition using phenylephrine. Our data indicate that nNOS plays a role in the rapidity of the initial rise in blood flow during NVC.

### Neuronal Nitric Oxide Synthase and Resting Steady-State Hemodynamics

The systemic hemodynamic changes seen following selective nNOS inhibition with SMTC are consistent with prior published data, showing an increase in systemic vascular resistance and MAP, with a minor decrease in cardiac output and heart rate.^14^ The rise in MAP to phenylephrine (as pressor control) was well matched to that induced by SMTC, while the differences in pattern of change in cardiac output and systemic vascular resistance are consistent with those observed in studies comparing phenylephrine with nonselective NOS inhibition using N(G)-monomethyl-L-arginine.^26^ Previous human studies assessing the role of NOS in regulation of cerebral blood flow have used N(G)-monomethyl-L-arginine, which, due to the nonselective action of this drug, provides little insight into the role of specific NOS isoforms.^27,28^ In rodents, selective nNOS inhibition with 7-nitroindazole reduced basal CBF,^29,30^ while in humans, selective nNOS inhibition with SMTC decreases global and regional CBF as measured by brain MRI arterial spin labeling.^13^

### Neuronal Nitric Oxide Synthase Plays a Role in the Early Phase of Neurovascular Coupling

**Figure 5.**
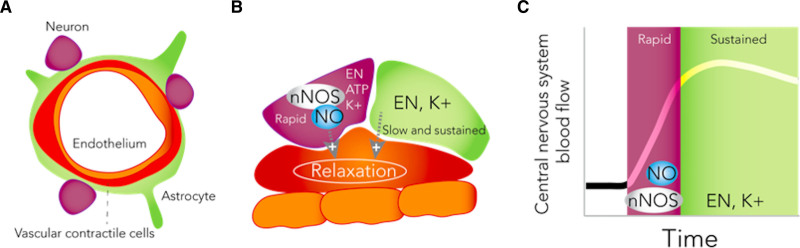
**Schematic representation of the potential role for neuronal nitric oxide synthase in neurovascular coupling in humans.** A, B, structure of the neurovascular unit. C, rise in regional cerebrovascular blood flow following stimulation consists of a rapid initial phase followed by a sustained phase. Neuronal activation initiates downstream cascades that promote NO production via nNOS, which affects the rapid initial phase of response. The sustained phase may be more dependent on eicosanoids (EN) and potassium (K+). Nitric oxide (NO), neuronal nitric oxide synthase (nNOS), adenosine triphosphate (ATP).

The rapidity of the initial rise in cerebral blood flow was reduced following nNOS inhibition, evidenced by traditional approaches for characterizing the early phase of the NVC response, (ie, the change in PCA flow velocity). Changes in newly developed markers of rapidity (iRate1 and iRate2) followed the same trend, but differences between conditions were not statistically significant. This is the first human study, complementing preclinical work, to show that nNOS plays a role in the early transient direct neuronal-vascular component of NVC.^30^ Continuous, near-instantaneous vascular responses are vital to meet temporal neuronal metabolic demands in the human brain, which lacks capacity for energy substrate storage. Our results are consistent with data from the rat brain, suggesting that NO levels rise early in the NVC response (≈400 ms)^31^ and further supports the contention that although NO may be important for the early response, the steady-state elevation in blood flow during NVC may rely predominantly on NO-independent astrocytic inter-communication. Although data from anaesthetized animal models suggest a role for NO in steady-state elevation, in unanesthetized rats, NOS inhibition has no effect.^29,32,33^ The effect of nNOS on NVC in the early and direct phase of the response is consistent with a modulatory fine-tuning role rather than as the primary mediator of increases in blood flow. The latter role may be subserved by several additional neuronal sources of vasodilators, such as eicosanoids, adenosine, adenosine triphosphate (which may evoke an endothelial nitric oxide synthase response), and oxygen (Figure 5).

A recent study in healthy young humans showed that nonselective NOS inhibition reduced the peak NVC response by ~30%.^34^ As our findings show no effect on peak NVC responses when using an established selective nNOS inhibitor, this previous work may suggest that peak NVC responses are due not to nNOS but other NOS isoforms, such as endothelial nitric oxide synthase. However, this deductive rationale should be interpreted with caution as the nonselective NOS inhibition condition was not compared with a matched pressor condition (+4 versus +15 mm Hg).^34^ More work is needed to consolidate these previous findings with our results.

### Implications

Dysfunctional NVC has been identified in a range of neurological conditions. In a preclinical model of Alzheimer’s dementia, tau induces dissociation of nNOS from postsynaptic density protein 95 (PSD95) in the post-synaptic neuron, impairing NVC.^35^ This points to a role for NVC integrity as a disease biomarker, and also potentially identifies nNOS as a therapeutic target capable of improving function by enhancing NVC. Moreover, increasing nNOS activity may mitigate central nervous system hypoperfusion and hypoxia, such as that preventing neurological recovery in the acute phase of spinal cord injury.

A relatively small increase in mean arterial pressure with phenylephrine, of approximately 10 mm Hg, increased NVC. This observation is aligned with our previous work showing that NVC is highly sensitive to changes in perfusion pressure.^2,17^ This consistent finding should be taken into consideration when interpreting prior studies and future study designs.^34,36,37^

In this study, we hypothesize that the effects of nNOS in NVC are due to NO’s effect on vascular smooth muscle. However, this does not fully appreciate the complexity of downstream NO signaling. For example, NO inhibits cytochrome p450 and may therefore decrease cytochrome p450-incuded production of the potent vasoconstrictor 20-HETE from arachidonic acid,^38^ therefore providing another mechanism whereby local NO production may promote an enhanced NVC response.^39,40^ The interaction between nNOS and 20-HETE (and other signaling molecules) in NVC is therefore a key area for future research.

### Limitations

This study had a relatively small sample size of healthy volunteers, and as such the findings cannot be extrapolated to disease states without further study in specific patient groups. In female participants, we did not control for phase of the menstrual cycle. Consistent with a noninvasive study in healthy volunteers, several of the outcome variables are indirect estimates and not direct measures of NVC. Using transcranial Doppler to estimate cerebral blood flow assumes consistent cross-sectional area of the insonated vessel. We chose to insonate the P1 to mitigate potential changes in PCA diameter during visual stimulation.^41^ Previous human work has shown that local infusion of SMTC did not affect diameter of radial artery, and had only a small effect on basal epicardial/conduit vessel tone.^19^ However, the effect of SMTC on cerebral artery diameter is unknown, and should be addressed by future studies. We used phenylephrine as a pressor control condition; however, we are unable to rule out the possibility that some or all of the effect seen is by mechanisms other than change in cerebral perfusion pressure due to increased MAP (eg, a direct effect of phenylephrine on the conduit cerebral arteries).

## Conclusion

Neuronal NOS plays a fundamental physiological role in the regulation of cerebral blood flow, particularly the rapidity of the NVC response to visual stimulation. The role of nNOS in conditions of impaired NVC warrants further evaluation.

## Article Information

### Acknowledgments

We are grateful to Karen McNeill and the staff at the Biomedical Research Centre, Clinical Research Facility, Guy’s and St Thomas NHS Foundation Trust for supporting this study and to the team from Guy’s Hospital Aseptic Pharmacy for the production of SMTC. We are grateful to Dr David Vickers PhD, University of Calgary for his statistical advice.

### Sources of Funding

Funding: The laboratory of A.A. Phillips is supported by the Canadian Institutes for Health Research (Project Grant), Brain Canada, Compute Canada, Natural Sciences and Engineering Research Council (Canada; Discovery Grant), the Libin Cardiovascular Institute of Alberta, Hotchkiss Brain Institute, Campus Alberta Neuroscience, and Rick Hansen Institute. This work was supported by the National Institute for Health Research Biomedical Research Centre (NIHR BRC) at Guy’s & St Thomas’ NHS Foundation Trust and King’s College London [IS-BRC-1215-20006]. We also acknowledge support from the British Heart Foundation [CH/1999001/11735, RE/18/2/34213 to AMS]; and a UK Medical Research Council Clinical Research Training Fellowship [MR/R017751/1] to KOG. Funding sources had no involvement in the study design, the collection, analysis, and interpretation of data, the report writing, or the decision to submit for publication.

### Disclosures

The iNVC software used in the data analysis is commercially available and was developed by Lam and Phillips.

### Supplemental Materials

Tables S1–S4

Figures S1–S4

Major Resources Table

## Supplementary Material
