# Supplementary material for: The Effect of a Neuronal Nitric Oxide Synthase Inhibitor on Neurovascular Regulation in Humans
Source: Circ Res. 2022 Nov 9;131(12):952–61. doi: 10.1161/CIRCRESAHA.122.321631 (PMC9770134; doi:10.1161/CIRCRESAHA.122.321631)
Supplement: Supplementary file 1 [file res-131-0952-s001.pdf]

Supplemental Materials

The effect of a neuronal nitric oxide synthase inhibitor on neurovascular regulation in humans

#Kevin O'Gallagher[1,2], #Ryan E. Rosentreter[3], Jan Elaine Soriano[3], Ali Roomi[1,2], Saqib Saleem[4], Tyler Lam[3], Roman Roy[1], Grant R. Gordon[3], Satish R. Raj[3], Philip J. Chowienczyk[1,2], &Ajay M. Shah[1,2†], &Aaron A. Phillips[3†]

[1] School of Cardiovascular and Metabolic Medicine & Sciences, King's College London British Heart Foundation Centre of Research Excellence, London, UK.

[2] NIHR Biomedical Research Centre, Clinical Research Facility, Guy's and St Thomas NHS Foundation Trust, London, UK.

[3] Departments of Physiology and Pharmacology, Clinical Neurosciences, Cardiac Sciences, Hotchkiss Brain Institute, Libin Cardiovascular Institute of Alberta, Cumming School of Medicine, University of Calgary, Alberta, Canada.

[4] Department of Electrical and Computer Engineering, COMSATS University, Sahiwal, Pakistan.

# Equal contribution
& Equal contribution

† Correspondence:
Aaron A. Phillips, aaron.phillips@ucalgary.ca, (403) 220-5672, Heritage Medical Research Building 93, 3330 Hospital Dr NW, Calgary, AB, Canada, T2N 4N1.

Ajay M. Shah, ajay.shah@kcl.ac.uk, 0044 20 7848 5189, The James Black Centre, 125 Coldharbour Lane, London, SE5 9NU, United Kingdom.

Contents

Table S1. Participant characteristics

Table S2. NVC results

Table S3. Coefficient of Variation data

Table S4. Shapiro-Wilk Normality data

Figure S1. Study Participation

Figure S2. Test-retest reliability

Figure S3. Analysis of sex-based differences

Figure S4. Analysis of association between haemodynamic conditions and NVC effects.

Major Resources Table

Participant number	Age	Weight (kg)	Height (cm)	Sex	Ethnicity
001	36	63	165	F	Caucasian
002	26	70	178	M	Caucasian
004	24	65	160	F	Caucasian
007	42	70	167	F	South Asian
009	18	65	165	F	Caucasian
010	28	73	178	M	Caucasian
011	24	56	165	F	Caucasian
012	21	54	161	F	East Asian
013	25	53	160	F	East Asian
014	34	89	174	M	South Asian
015	26	64	173	M	Caucasian
016	25	82	180	M	East Asian
Mean (SD)	27.4 (6.7)	67.0 (10.8)	168.8 (7.4)	(7 F, 5M)	

Table S1 Participant characteristics

	Placebo control		SMTC		Pressor control	
	**Pre**	**Post**	**Pre**	**Post**	**Pre**	**Post**
MAP (%)	-0.23±0.4	0.15±0.5	0.17±0.6	-0.68±0.4	0.37±0.6	0.67±0.3
Mean PCAv (%)	8.1±0.6	8.8±0.5	7.6±1.0	6.7±1.0	6.82±0.6	8.10±0.7
Peak PCAv (%)	19.0±0.7	20.9±1.1	19.1±2.0	17.0±1.9	17.4±1.3	19.5±1.7
PCA PI (%)	-1.9±0.6	-2.2±0.5	-2.1±0.8	-2.5±0.9	-1.9±1.0	-3.1±0.6
PCA PR (%)	-3.5±0.5	-2.4±0.8	-3.0±0.5	-2.1±0.4	-3.1±0.6	-3.0±0.5
Time to peak (s)	18.8±2.4	16.2±2.4	19.1±1.9	15.5±2.6	15.4±2.1	14.1±2.2
Time to max slope (s)	5.7±0.7	7.3±1.7	5.5±0.5	6.1±0.5	5.3±0.2	4.9±0.1
Mean PCAv 0-5s (%)	5.5±0.8	7.2±0.5	5.6±0.9	4.5±0.9	4.7±0.8	7.3±0.8
Mean PCAv 5-10s (%)	13.1±0.9	15.2±0.9	13.1±1.8	12.2±1.6	12.7±1.2	16.2±1.4
Mean PCAv slope, 0-5s (%)	11.4±1.0	13.5±1.0	11.4±1.0	13.5±1.0	11.2±1.2	14.0±1.2
Max PCAv slope, 0-5s (%)	10.1±0.7	11.5±1.0	9.8±1.2	7.4±1.2	9.2±0.8	11.3±1.1
Minimum PCAv slope, 0-5s (%)	12.9±1.6	15.7±1.5	14.9±2.2	13.0±2.6	13.5±2.4	17.2±1.9

Table S2 NVC Results

PI, pulsatility index; PR, pulse ratio; PCAv, posterior cerebral artery velocity. Data expressed as mean±SEM

Subject	Placebo control	SMTC	Pressor control	**Subject mean**
1	7.59%	8.03%	7.51%	**7.71%**
2	9.69%	10.05%	9.15%	**9.63%**
4	6.66%	8.76%	6.95%	**7.46%**
7	5.54%	6.28%	8.03%	**6.62%**
9	8.34%	7.22%	5.40%	**6.99%**
10	10.25%	10.69%	9.74%	**10.23%**
11	5.25%	4.89%	6.72%	**5.62%**
12	6.37%	6.12%	5.59%	**6.03%**
13	8.13%	6.61%	8.27%	**7.67%**
14	6.07%	6.62%	6.22%	**6.30%**
16	9.79%	4.72%	5.36%	**6.62%**
**Condition mean**	**7.61%**	**7.27%**	**7.18%**	

Table S3 Coefficient of Variation Data

Variable	Placebo Control	SMTC	Pressor Control
ΔAverage PCA velocity (cm/s)	W= 0.94<br>P= 0.53	W= 0.93<br>P= 0.40	W= 0.93<br>P= 0.38
ΔAverage MCA velocity (cm/s)	W= 0.92<br>P= 0.34	W= 0.92<br>P= 0.35	W= 0.97<br>P= 0.85
ΔPCA CVR (mmHg/cm*s^{-1})	W= 0.91<br>P= 0.26	W= 0.82<br>P= 0.020	W= 0.96<br>P= 0.73
ΔMCA CVR (mmHg/cm*s^{-1})	W= 0.86<br>P= 0.060	W= 0.61<br>P= $3.1x10^{-5}$	W= 0.94<br>P= 0.50
ΔAverage PCA velocity 0-5s (%)	W= 0.95<br>P= 0.70	W= 0.94<br>P= 0.57	W= 0.96<br>P= 0.73
ΔAverage PCA velocity slope 0-5s (%)	W= 0.97<br>P= 0.86	W= 0.96<br>P= 0.77	W= 0.94<br>P= 0.49
ΔTime of max PCA slope	W= 0.90<br>P= 0.21	W= 0.83<br>P=0.031	W= 0.92<br>P= 0.38
ΔiRate 1	W= 0.98<br>P= 0.97	W= 0.96<br>P= 0.82	W= 0.94<br>P= 0.55
ΔiRate 2	W= 0.94<br>P= 0.51	W= 0.96<br>P= 0.81	W= 0.97<br>P= 0.85
ΔiAmp 1	W= 0.97<br>P= 0.90	W= 0.92<br>P= 0.30	W= 0.95<br>P= 0.61
ΔiAmp 2	W= 0.93<br>P= 0.36	W= 0.94<br>P= 0.48	W= 0.96<br>P= 0.73
ΔiPulse	W= 0.79<br>P= 0.0070	W= 0.84<br>P= 0.030	W= 0.97<br>P= 0.89
ΔPCA CVCi (cm/s/mmHg)	W= 0.84<br>P=0.031	W=0.85<br>P=0.037	W= 0.77<br>P= 0.0041
ΔPCA CVCi (%)	W= 0.95<br>P= 0.70	W= 0.93<br>P= 0.43	W= 0.91<br>P= 0.22
ΔNVC MAP (%)	W= 0.86<br>P= 0.064	W= 0.92<br>P= 0.32	W= 0.90<br>P= 0.16

Table S4 Shapiro-Wilk Normality Data

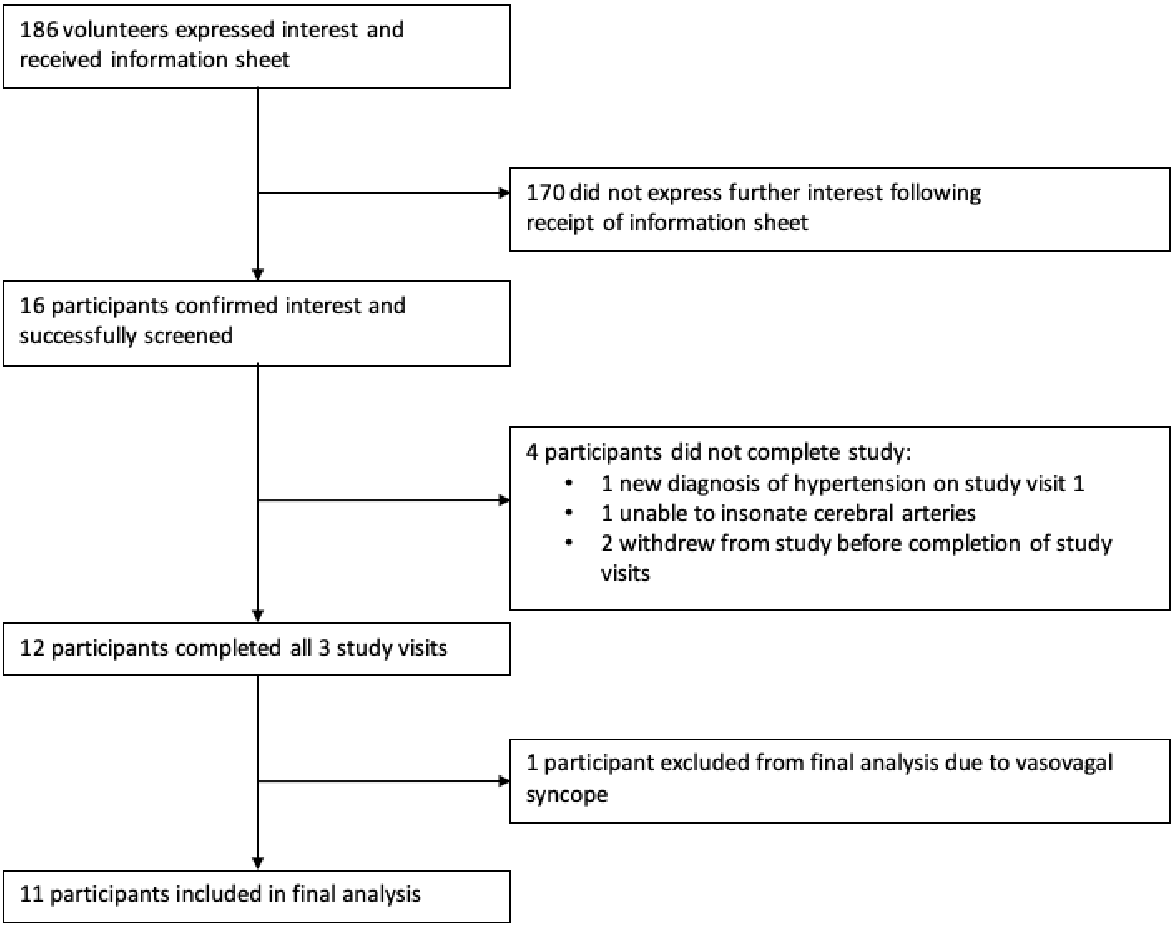

Figure S1. Study Participation

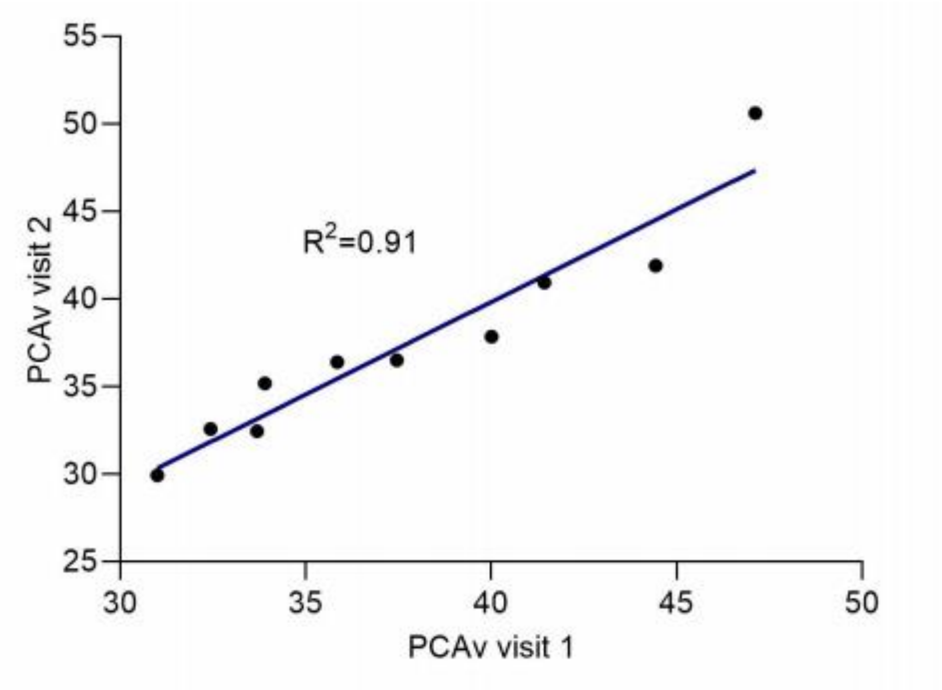

Figure S2. Test-retest reliability

Here we show our test-retest reliability for baseline PCA velocity values taken from 10 participants tested on 2 separate days, a minimum of 1 week apart. The evaluation of test-retest reliability using an intraclass correlation coefficient (ICC) is the preferred metric of use.[49] We report an ICC score of 0.951.

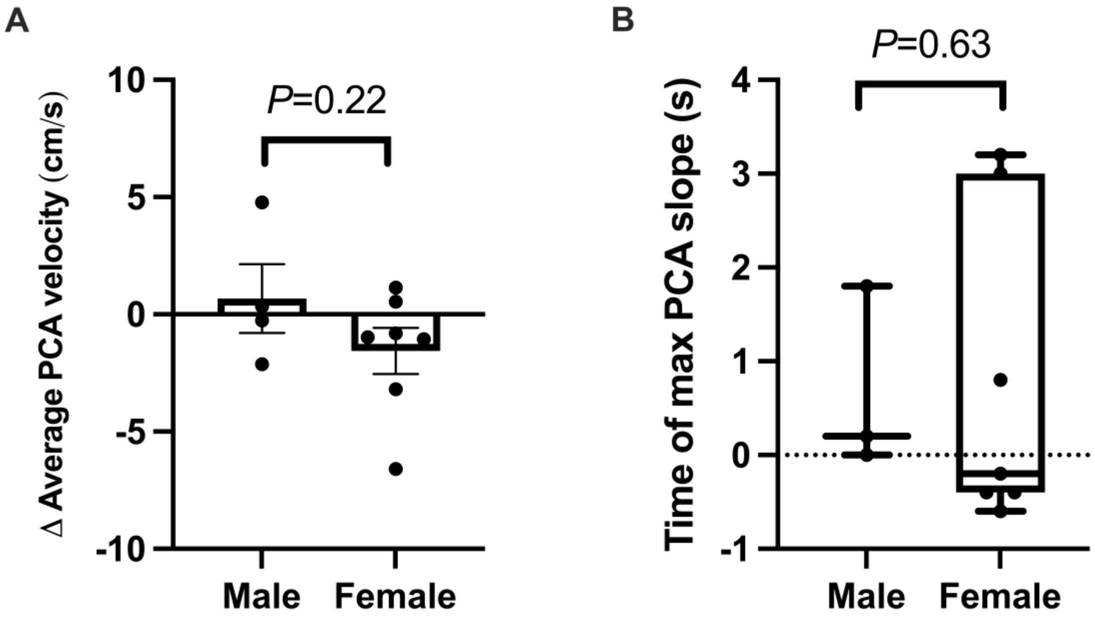

Figure S3. Analysis of sex-based differences in PCA responses to SMTC, as analysed by unpaired t test (panel A, mean±SEM) or Mann-Whitney test (panel B, median (IQR)). n=11 for panel A (4 male, 7 female), n=10 for panel B (3 male, 7 female).

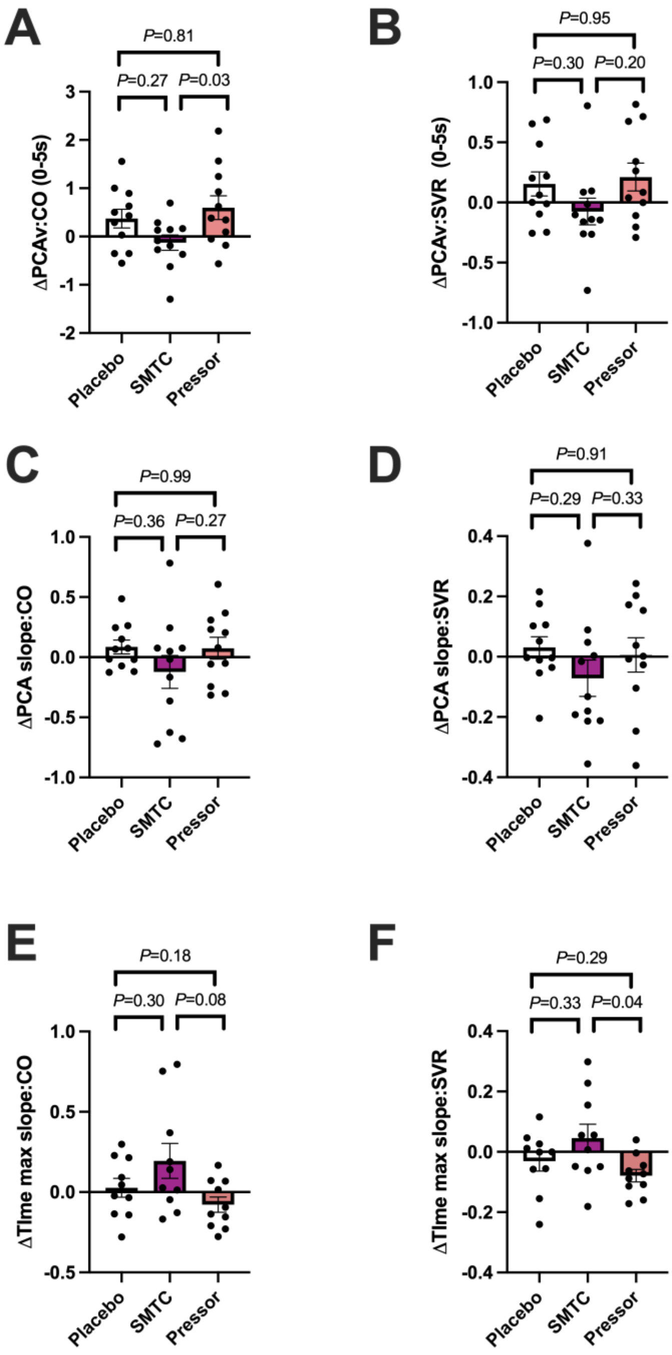

Figure S4. Changes in neurovascular coupling velocity corrected for changes in haemodynamic conditions. Panels A, C, E show indices corrected for CO**.** Panels B, D, F show indices corrected for SVR. Analysis performed using one way ANOVA and Tukey *post hoc*. Data presented as mean±SEM. n=11, apart from panels E&F (n=10).

Major Resources Table

Description	Source / Repository	Persistent ID / URL
Study drug: S-methyl-L-thiocitrulline	Merck Millipore, USA	
Study drug: Phenylephrine	Amdipharm UK Ltd	
Intellivue Brachial blood pressure monitor	Phillips, UK	
Finometer NOVA Finger plethysmograph	Finapres Medical Systems, The Netherlands	
Transcranial Doppler probes: DWL DopplerBox X	Compumedics, Singen, Germany	
QL Monitoring Software, version 3.5.5		
RespirAct	Thornhill Research, Toronto, Canada	
Tegaderm transparent dressing film	3M Healthcare, St Paul, Minnesota, USA	
iNVC Software, Version 2.2	Innovate Calgary, Alberta, Canada	
